# Digital health interventions for family caregivers of stroke survivors: a scoping review of caregiver outcomes, implementation factors, and evidence gaps

**DOI:** 10.3389/fdgth.2026.1938198

**Published:** 2026-10-14

**Authors:** Supriadin, Regidor III Poblete Dioso, Hafizah Che Hassan

**Affiliations:** 1 School of Nursing & Applied Science, Lincoln University College, Petaling Jaya, Selangor D E, Malaysia; 2 Nursing Study Program, Faculty of Public Health, STIKES Yahya Bima, West Nusa Tenggara, Indonesia

**Keywords:** caregiver, digital health, mHealth, quality of life, scoping review, stroke, telehealth

## Abstract

**Background:**

Stroke is a leading cause of long-term disability and often transfers substantial care responsibilities to family and informal caregivers. These demands contribute to multidimensional caregiver burden and reduced quality of life (QoL), including psychological distress, social limitations, and financial strain. Digital health interventions—such as mobile applications, messaging-based education, telehealth, and web-based platforms—have the potential to extend caregiver support beyond conventional face-to-face services; however, evidence regarding their impact on caregiver QoL remains heterogeneous. This scoping review aimed to map and characterize digital health used in stroke caregiving and to summarize their associations with caregiver QoL–related outcomes, including caregiver burden, psychological well-being, empowerment or capability, usability, and access.

**Methods:**

A scoping review was conducted in accordance with Joanna Briggs Institute (JBI) guidance and reported following PRISMA-ScR. Searches were performed in PubMed, Scopus, Web of Science, CINAHL, and Google Scholar for English-language studies published between 2019 and 2025. Two reviewers independently screened studies and extracted data using a standardized charting form. Evidence was mapped descriptively by intervention type, delivery characteristics, study design maturity, and caregiver outcome domains.

**Results:**

From 676 identified records, 20 studies met the inclusion criteria. Digital interventions were primarily delivered through mobile applications, WhatsApp-based education, telehealth services, or web-based learning platforms. Direct caregiver-focused studies commonly assessed caregiver burden, psychological distress, and caregiving capability, while system-integrated mHealth programs mainly reported patient outcomes with indirect relevance to caregivers. Overall, digital education and follow-up support were associated with reduced caregiver burden and improved caregiver capability and emotional well-being, although outcome measures and follow-up durations varied. Usability, digital literacy, affordability, and connectivity were recurrent barriers.

**Conclusion:**

Digital health interventions may support caregiver preparedness, burden reduction, psychological well-being, and access to support; however, evidence regarding effectiveness remains uncertain due to heterogeneity in intervention design, outcome measurement, and study quality.

## 1 Introduction

Stroke is a leading cause of long-term disability worldwide and commonly results in persistent functional limitations that shift substantial responsibilities to family and informal caregivers. The cumulative demands of assisting with mobility, activities of daily living, medication management, secondary prevention, and emotional support place caregivers at heightened risk of multidimensional burden and deteriorating quality of life (QoL), including psychological distress, social restriction, and financial strain (1, 2). Caregiving outcomes are shaped not only by patient severity and rehabilitation needs but also by caregiver resources, self-efficacy, and access to reliable support systems, which can vary markedly across settings and socioeconomic contexts (3, 4).

Digital health interventions, including mobile health (mHealth) applications, messaging-based education (e.g., WhatsApp), telehealth, and web-based learning platforms such as portals and massive open online courses (MOOCs), have emerged as scalable approaches to extend caregiver support beyond face-to-face services. These technologies can deliver structured education, skills training, reminders, monitoring, and follow-up contact, potentially strengthening caregiver capability and confidence while reducing caregiving strain (2, 5, 6). Across stroke-related digital programs, feasibility and acceptability have been frequently reported as high, supported by retention, adherence, and satisfaction indicators in pilot and feasibility trials (7, 8). Importantly, system-integrated mHealth models delivered through primary care have demonstrated sustained clinical benefits at the patient level, including improved risk factor control and longer-term outcomes, suggesting that digital approaches can be effective when embedded within routine care pathways (9, 10).

However, the potential of digital interventions to improve caregiver QoL is constrained by implementation conditions and user experience. Qualitative evidence indicates that caregivers and older stroke survivors value continuity of guidance, trustworthy information, and age-tailored features (e.g., simplified interfaces and reminders), but also express concerns about privacy, affordability, and the credibility of app content (1, 4). In addition, large-scale user review analyses of caregiving apps highlight recurring problems related to usability, accessibility, and perceived value that may undermine sustained engagement and real-world impact (11). Studies of technology readiness and digital adoption further suggest that digital literacy, training access, and contextual barriers such as cost and connectivity can widen inequities if interventions are not designed and implemented inclusively (12). From the perspective of implementation stakeholders, healthcare professionals often report strong willingness to adopt caregiver-facing mHealth tools, while emphasizing the need for caregiver-relevant service features and workflow-compatible designs (13).

Given the rapid growth and heterogeneity of digital caregiver support strategies ranging from education via messaging platforms to web-based capability-building, dyadic self-care programs, and system-level models, there remains a need to comprehensively map what types of digital interventions have been studied, which caregiver QoL domains have been measured, and what mechanisms (e.g., empowerment, usability, and access) appear to shape outcomes. Scoping review methodology is appropriate to address this objective by systematically identifying, charting, and synthesizing diverse evidence, including trials, feasibility studies, qualitative studies, and economic evaluations (14).

Although digital health interventions have expanded in stroke care, existing research has predominantly focused on patient outcomes, rehabilitation performance, and clinical indicators. The extent to which these interventions directly support family caregivers and improve caregiver-centered outcomes remains unclear. Furthermore, caregiver outcomes are frequently treated as secondary outcomes, making it necessary to distinguish caregiver-directed interventions from patient-focused interventions with indirect caregiver implications.

Accordingly, this scoping review aimed to map evidence on digital health interventions for stroke caregiving and their association with caregiver QoL–related outcomes, including caregiver burden, psychological wellbeing, empowerment/capability, usability, and access to support.

## 2 Methods

### 2.1 Study design

This scoping review was conducted in accordance with the Joanna Briggs Institute (JBI) methodology for scoping reviews and is reported following the Preferred Reporting Items for Systematic Reviews and Meta-Analyses extension for Scoping Reviews (PRISMA-ScR) guidelines. The JBI framework provides a systematic approach for mapping the breadth and nature of evidence in emerging or complex research areas, including the identification of key concepts, sources, and knowledge gaps (15, 16). The PRISMA-ScR checklist was applied to enhance transparency, methodological rigor, and reproducibility in reporting the review process (14). Scoping reviews are particularly appropriate for this purpose because they allow comprehensive exploration of heterogeneous evidence across diverse digital health modalities, such as mobile applications, telehealth platforms, and web-based caregiver support systems (15, 16). The review protocol was developed *a priori* and followed JBI guidance for scoping reviews. Although the protocol was not formally registered, all methodological steps were predefined before data extraction. The protocol was not registered in PROSPERO because scoping reviews are generally not eligible for registration in this database. However, methodological decisions were established before study screening and data extraction.

### 2.2 Eligibility criteria

Eligibility criteria were defined using the Population–Concept–Context (PCC) framework (Table 1).

**Table 1:** Eligibility criteria framework for study inclusion.

Domain	Description
Population	Family or informal caregivers of stroke survivors (e.g., spouses, adult children, relatives, or friends). Studies including stroke survivors or healthcare professionals were eligible only if caregiver-relevant outcomes, needs, or usability data were reported.
Concept	Digital health interventions intended to support stroke caregiving, including mobile health (mHealth) applications, messaging-based education (e.g., WhatsApp), telehealth services, web portals, massive open online courses (MOOCs), and other technology-enabled caregiver support programs.
Context	Community, primary care, rehabilitation, and hospital-to-home transition settings in any country.
Caregiver Outcome Requirement (Direct Evidence)	Studies were classified as providing direct caregiver-outcome evidence if they quantitatively measured at least one caregiver-relevant outcome (e.g., caregiver burden, caregiver quality of life, depression, anxiety, stress, self-efficacy, empowerment, caregiving competence, or social support) or evaluated caregiver-focused usability and engagement outcomes where caregivers were the primary end users.
Caregiver Outcome Requirement (Indirect Evidence)	Studies were classified as indirect caregiver-relevant evidence if they evaluated digital interventions primarily targeting stroke patients or health systems but included findings clearly relevant to caregivers (e.g., reduced hospital visits, improved continuity of care, caregiver involvement features), without directly measuring caregiver outcomes.
Study Designs	Quantitative, qualitative, mixed-methods, economic evaluations, and study protocols were eligible to map evidence maturity and intervention development stages.
Exclusion Criteria	Editorials, commentaries, and studies without caregiver relevance or caregiver-focused outcomes.
Language and Timeframe	English-language studies published between 2019 and 2025.

### 2.3 Information sources and search strategy

A comprehensive search was conducted in PubMed, Scopus, Web of Science, CINAHL (EBSCOhost), and Google Scholar between October and November 2025. Search terms combined controlled vocabulary (e.g., MeSH in PubMed) and keywords related to stroke, caregivers, digital health, and caregiver outcomes (e.g., “stroke caregiver*,” “family caregiver*,” “mHealth,” “telehealth,” “mobile app*,” “WhatsApp,” “web-based,” “quality of life,” “caregiver burden,” “wellbeing,” “self-efficacy,” “empowerment”). The complete search strategies for each database, including filters and field tags, are provided in Supplementary Material 1.

Google Scholar was searched using predefined keyword strings with results sorted by relevance. The first 200 results were screened because relevance declines substantially beyond early pages; duplicates and non-peer-reviewed items were removed during full-text review. The date searched and exact query strings are reported in Supplementary Material. No grey literature sources were included because the review aimed to map peer-reviewed evidence on digital caregiver interventions. Citation chaining was performed by screening reference lists of included studies and relevant reviews.

### 2.4 Source selection

All retrieved records were imported into a reference management system, and duplicates were removed. Two reviewers independently screened titles and abstracts against the eligibility criteria. Full-text articles were then assessed for final inclusion. Discrepancies were resolved through discussion until consensus was achieved. A PRISMA-ScR flow diagram was used to document the selection process, including the number of records identified, screened, excluded, and included in the final synthesis. Screening was performed using [Rayyan/Excel/EndNote]. Disagreements were resolved through discussion, and unresolved disagreements were reviewed by a third researcher.

### 2.5 Data charting process

A standardized data charting form was developed to systematically extract key methodological and outcome-related information from each included study. The extracted variables comprised the author and year of publication, study design, sample characteristics, instruments used, type of digital intervention, and main findings related to caregiver outcomes. Data extraction was conducted independently by two reviewers to ensure accuracy, consistency, and completeness of the recorded information.

### 2.6 Synthesis of results

Results were mapped descriptively and thematically. Evidence was organized into direct caregiver-outcome evidence (quantitative caregiver measures and/or caregiver usability outcomes) and indirect caregiver-relevant evidence (patient/system programs with caregiver-relevant implications). Themes were synthesized across five domains: caregiver burden, psychological well-being, empowerment/capability, usability/user experience, and access/digital equity. Because this was a scoping review, findings were mapped rather than pooled, and causal language was avoided where evidence was preliminary or protocol-based.

### 2.7 Critical appraisal and ethics

Consistent with the objectives of a scoping review, no formal critical appraisal of study quality was conducted, as the focus was on mapping existing evidence rather than assessing intervention effectiveness. Ethical approval was not required, as this study synthesized data from previously published literature. Therefore, findings should be interpreted as evidence mapping rather than confirmation of intervention effectiveness.

## 3 Results

### 3.1 Searching results

A comprehensive database search identified 676 records. After removal of duplicates, 507 records underwent title and abstract screening. Of these, 419 articles were excluded because they did not meet the eligibility criteria, primarily due to the absence of a stroke caregiver population, lack of a digital health intervention, or failure to report caregiver-relevant outcomes. A total of 88 full-text articles were assessed for eligibility, and 68 studies were excluded mainly because interventions were not caregiver-relevant, caregiver outcomes were unavailable, or digital components were insufficiently described. Finally, 20 studies were included in this scoping review (Figure 1).

**Figure 1:**
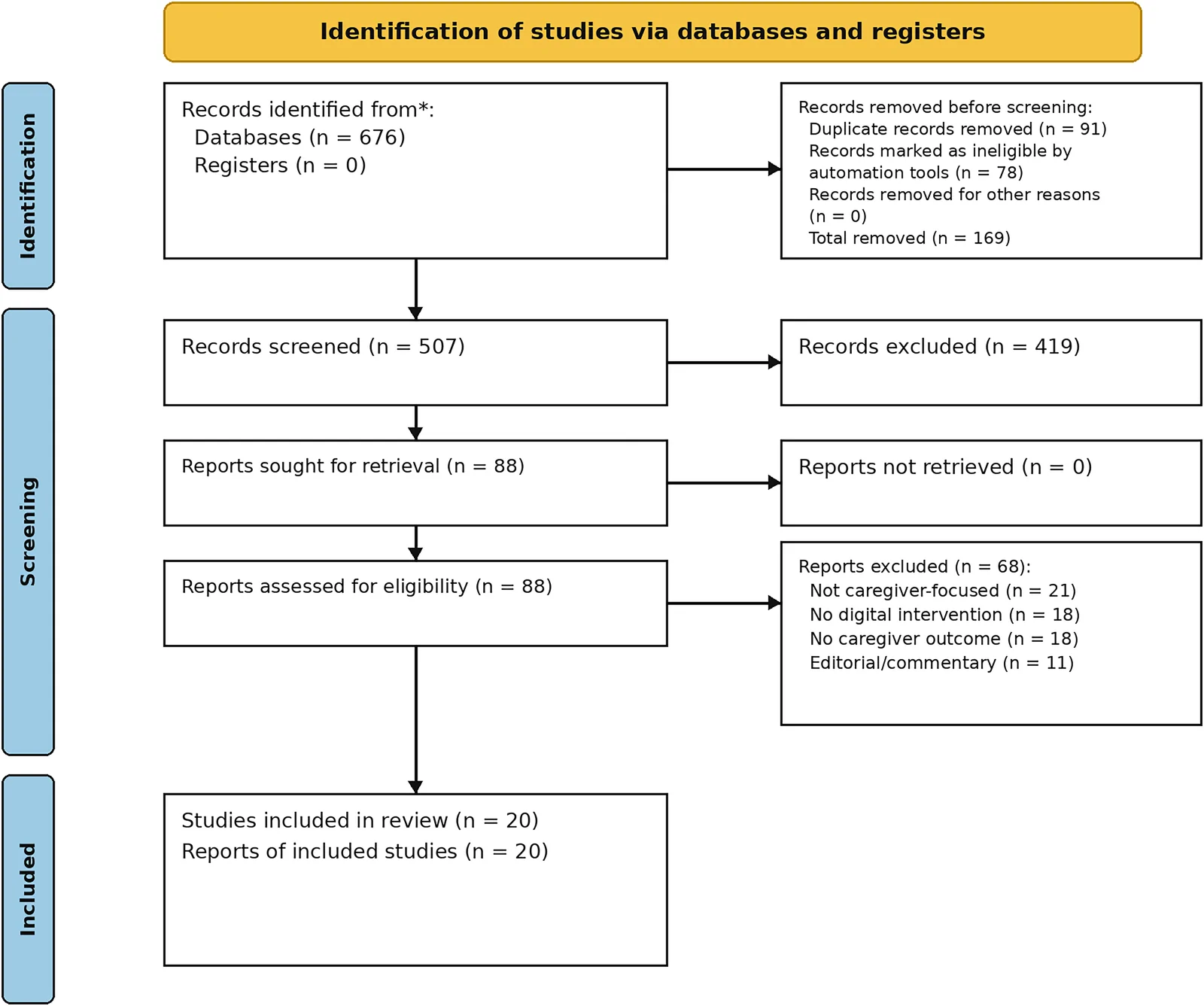
PRISMA-ScR flow diagram of study selection.

The included studies were mapped rather than pooled because of substantial heterogeneity in intervention modalities, target users, study designs, outcome measures, and follow-up periods. Therefore, the findings represent an evidence map of emerging digital health approaches and their potential relevance to caregiver-centered outcomes rather than an estimate of intervention effectiveness.

### 3.2 Characteristics of included studies

The 20 included studies demonstrated considerable variation in study design, geographical setting, intervention maturity, digital platforms, and caregiver involvement. Studies included randomized controlled trials, feasibility studies, qualitative studies, mixed-methods studies, economic evaluations, and intervention protocols, reflecting different stages of digital health development.

To improve conceptual clarity, studies were categorized according to the primary relationship between the digital intervention and caregiver outcomes:

(1) Direct caregiver-focused interventions:
  Studies in which caregivers were the primary target users and caregiver outcomes were directly measured. These interventions included caregiver education platforms, mobile applications, online learning programs, telehealth-based caregiver support, and dyadic interventions involving stroke survivors and caregivers.
(2) Patient-directed interventions with caregiver involvement:
  Studies primarily targeting stroke survivors but involving caregivers as partners in rehabilitation, self-management, or behavioral change activities.
(3) Indirect caregiver-relevant interventions:
  Studies focused mainly on patient outcomes or health-system outcomes, where potential caregiver benefits were inferred through improved continuity of care, reduced healthcare utilization, or increased caregiver participation, although caregiver outcomes were not directly measured.

Digital intervention modalities included mobile health applications, messaging-based education platforms (such as WhatsApp), web-based learning systems, massive open online courses (MOOCs), telehealth services, remote monitoring programs, and integrated primary-care mHealth systems. Intervention intensity ranged from self-guided educational resources to clinician-supported programs involving nurses, rehabilitation professionals, or healthcare teams.

Across studies, caregiver outcomes were measured using heterogeneous instruments, including caregiver burden scales, psychological well-being measures, self-efficacy assessments, usability questionnaires, and quality-of-life-related measures. Detailed characteristics of included studies, including intervention target, platform, duration, outcome instruments, and key findings, are presented in Table 2.

**Table 2:** Characteristics of included studies and caregiver-relevant outcomes (direct vs. Indirect Evidence).

Author (Year)	Country/Setting	Design Maturity	Participants (Caregiver Type, n)	Intervention Modality	Duration & Human Support	Caregiver Outcomes Measured?	Caregiver Instruments	Key Caregiver Findings	Implementation/Usability/Access Notes
(Sidek et al., 2023) (13)	Malaysia/healthcare system	Mixed methods	HCPs (*n*=239); caregivers indirect	mHealth app for stroke caregiver support	Not specified; HCP-guided	Indirect	TAM questionnaire	High intention to use; perceived caregiver skill improvement	Need for caregiver-focused features; high acceptability
(Thurston et al., 2025) (5)	Sweden/community	Feasibility RCT	Patients (*n*=114); caregivers indirect	mHealth PA + behavior change	6 months; clinician supervised	Indirect	None	Improved feasibility, adherence	Mild adverse events; high app satisfaction
(Kumar et al., 2024) (6)	India/home care	Feasibility	Dyads (*n*=30)	Web-based Stroke Home Care app	Not specified; education support	Direct	CVI; satisfaction	Potential burden reduction; high satisfaction	Good content validity; feasible
(Haji Mukhti et al., 2022) (1)	Malaysia/rehab	Qualitative	Informal caregivers (*n*=13)	Needs assessment for mobile app	NA; interview-based	Direct	Interview guide	Desire for app to reduce burden	Need for info access; cost concerns
(Fuller-Tyszkiewicz et al., 2020) (19)	Australia/online	RCT	Caregivers (*n*=183)	Psychological self-guided app	5 weeks; no human support	Direct	DASS; wellbeing scales	Stress ↓; wellbeing ↑ (3–4 months)	High engagement; scalable
(Canto et al., 2025) (17)	Brazil/community	Protocol	Caregivers (planned *n*=58)	Virtual education + calls	Not started; nurse follow-up	Direct (planned)	Burden, knowledge	Outcomes pending	Feasibility focus
(Nazari et al., 2024) (2)	Iran/hospital-home	RCT	Dyads (*n*=60)	WhatsApp education	15 sessions; nurse-led	Direct	ZBI; SS-QOL	Burden ↓; QoL ↑ post	Good engagement; connectivity
(Zainal et al., 2025) (21)	Singapore/community	Qualitative	Informal caregivers (*n*=30)	Digital health experiences	NA; interview	Direct	Interview guide	Barriers to tech use	Low digital literacy; cost
(Cao et al., 2025) (4)	China/hospital	Qualitative	Older survivors; caregivers indirect	Stroke app perspectives	NA	Indirect	Interviews	Need for tailored apps	Privacy; usability concerns
(Lobo et al., 2023) (11)	Global/app users	UX analysis	App users (*n*=13,368)	Caregiving apps	NA	Indirect	UX text mining	Usability problems	Credibility; accessibility
(Hayashi et al., 2025)	Japan/community	RCT	Adults + families (*n*=101)	Nutrition app + family bubbles	3 months; family support	Indirect	Social frailty	Social behavior ↑	Family involvement helpful
(Yan et al., 2021) (10)	China/rural	Cluster RCT	Patients; caregivers indirect	SINEMA mHealth	12 months; primary care	Indirect	QoL scales	QoL ↑; recurrence ↓	System integration key
(Yang et al., 2025) (22)	China/rural	Economic	Patients; caregivers indirect	SINEMA cost-effectiveness	6 years	Indirect	QALYs	Highly cost-effective	Scalable
(Wang et al., 2023) (18)	China/hospital	Protocol	Dyads (planned *n*=152)	Dyadic Self-Care App	Not started; nurse-guided	Direct (planned)	ZBI; SS-QOL	Outcomes pending	Feasibility
(Bakas et al., 2021) (3)	USA/home	Mixed methods	Caregivers (*n*=22)	TASK III via telehealth	Variable; nurse-led	Direct	Satisfaction ratings	Prefer multi-platform	Personalization needed
(Chen et al., 2025) (9)	China/rural	Long-term follow-up	Patients; caregivers indirect	SINEMA	6 years	Indirect	Mortality	Mortality ↓	Long-term system impact
(Cardy et al., 2024) (20)	Ireland/rehab	Qualitative	Caregivers + HCPs (*n*=28)	Adaptive mHealth PA	NA	Indirect	Interviews	Support for mHealth	Implementation challenges
(Babkair et al., 2025) (12)	Saudi Arabia/community	Cross-sectional	Public (*n*=576)	Social media for stroke	NA	Indirect	TRI	High readiness	Age/education gaps
(Kringle et al., 2020) (7)	USA/community	Case series	Stroke survivors	iADAPTS app	5 weeks; therapist	Indirect	CSQ-8	High satisfaction	Feasible
(Costa et al., 2025) (5)	Brazil/community	RCT	Caregivers (*n*=58)	e-Share MOOC	Self-paced; minimal support	Direct	Caregiver Burden Scale	Burden ↓	Accessible education

### 3.3 Thematic findings

The synthesis identified six interconnected domains describing how digital health interventions may influence caregiver-centered outcomes:

1. Caregiver burden and quality-of-life-related outcomes
2. Psychological well-being and emotional support
3. Caregiver empowerment, knowledge, and capability
4. Usability and user experience
5. Access, digital literacy, and health equity
6. Implementation characteristics of digital interventions

The first three domains represent caregiver outcomes, whereas usability, access, and implementation characteristics represent mechanisms that may influence intervention engagement and potential benefit. Because included studies varied substantially in design and methodological rigor, findings are interpreted as evidence of potential benefit rather than definitive evidence of effectiveness.

#### 3.3.1 Caregiver burden and quality-of-life-related outcomes (direct evidence)

Studies directly evaluating caregiver outcomes suggested that digital education, structured follow-up support, and caregiver-focused applications may contribute to reductions in perceived caregiver burden and improvements in caregiving confidence. Interventions delivered through WhatsApp education, web-based learning platforms, and caregiver mobile applications commonly focused on stroke-related knowledge, medication management, complication prevention, and daily caregiving skills (2, 5, 6).

The most consistent improvements were observed in task-related caregiving capability rather than broad quality-of-life outcomes. For example, educational interventions improved caregiver preparedness and confidence in managing stroke-related care demands, while some studies reported reductions in caregiver burden scores. However, interpretation remains limited because studies differed in sample size, intervention duration, measurement instruments, and methodological design. Several included studies were feasibility studies or protocols, limiting conclusions regarding sustained long-term effects on caregiver quality of life (7, 17, 18).

#### 3.3.2 Psychological well-being and emotional support (direct evidence)

Several digital interventions demonstrated potential benefits for caregiver psychological outcomes, including reduced stress, improved emotional well-being, and increased perceived social support. Smartphone-based psychological interventions and digital support programs showed improvements in stress-related outcomes among caregivers; however, most interventions were not specifically developed for stroke caregivers and were frequently adapted from broader caregiver populations (19, 20).

Evidence regarding psychological outcomes remains limited because few studies incorporated validated mental health outcomes as primary endpoints. Future caregiver-centered digital interventions should prioritize psychological support components, particularly for caregivers experiencing prolonged caregiving demands, emotional distress, and social isolation.

#### 3.3.3 Caregiver empowerment, knowledge, and capability (direct evidence)

Educational and skills-based digital interventions consistently emphasized caregiver empowerment as a key pathway through which digital health may support caregiving. Programs providing structured information, self-management guidance, and professional follow-up were associated with improvements in caregiver knowledge, perceived competence, and confidence in performing caregiving tasks (5, 6, 17).

Healthcare professionals also reported that caregiver-oriented mHealth applications were acceptable and potentially useful for improving caregiver education and communication with healthcare providers (13). However, these findings primarily reflect feasibility, acceptability, and implementation readiness rather than confirmed long-term improvements in caregiver quality of life.

#### 3.3.4 Usability and user experience (implementation mechanism)

Usability emerged as a critical factor influencing engagement with digital caregiver interventions. Studies highlighted that caregivers preferred digital tools with simple navigation, clear instructions, clinically reliable information, and features adapted to caregiver needs and technological ability (1, 4).

Analysis of large-scale user reviews of caregiving-related mobile applications identified recurring concerns related to interface complexity, accessibility, credibility of information, and perceived usefulness (11). These findings suggest that digital interventions require user-centered design approaches, including caregiver involvement during development, usability testing, and adaptation to different levels of digital experience.

#### 3.3.5 Access, digital literacy, and health equity (implementation mechanism)

Access-related barriers were frequently reported across studies and included limited digital literacy, affordability concerns, inadequate device access, connectivity problems, and insufficient training. Digital readiness varied according to caregiver age, education level, socioeconomic status, and previous experience with technology (12).

Informal caregivers reported difficulties related to learning new technologies, navigating digital platforms, and maintaining engagement when technical support was unavailable (21). Concerns regarding privacy, trust, and credibility of online health information were also identified, particularly among older caregivers and users with limited digital experience (4).

These findings indicate that digital health interventions may unintentionally widen disparities if accessibility, affordability, and digital literacy are not incorporated into intervention design and implementation strategies. These findings indicate that digital health interventions may unintentionally widen disparities if accessibility, affordability, and digital literacy are not incorporated into intervention design and implementation strategies.

#### 3.3.6 Intervention characteristics associated With potential benefit

Across included studies, several intervention characteristics appeared to influence potential caregiver benefits.

First, interventions incorporating structured education combined with professional support generally demonstrated stronger caregiver engagement compared with purely informational resources. Second, interactive and personalized approaches, including nurse-guided communication, feedback mechanisms, and dyadic caregiver–patient involvement, appeared more aligned with caregiver needs than passive educational content alone.

Third, interventions integrated into existing healthcare pathways, such as primary care or rehabilitation services, demonstrated greater potential for sustainability because they combined digital access with human support. However, comparative evidence remains insufficient to determine whether synchronous, asynchronous, mobile-based, or web-based approaches are superior.

### 3.4 Conceptual model of digital health intervention pathways

The conceptual model illustrates potential pathways through which digital health interventions may influence caregiver-centered outcomes (Figure 2). Digital tools, including mobile applications, messaging-based education, telehealth, and web-based platforms, provide caregivers with information, skills training, communication opportunities, and ongoing support.

**Figure 2:**
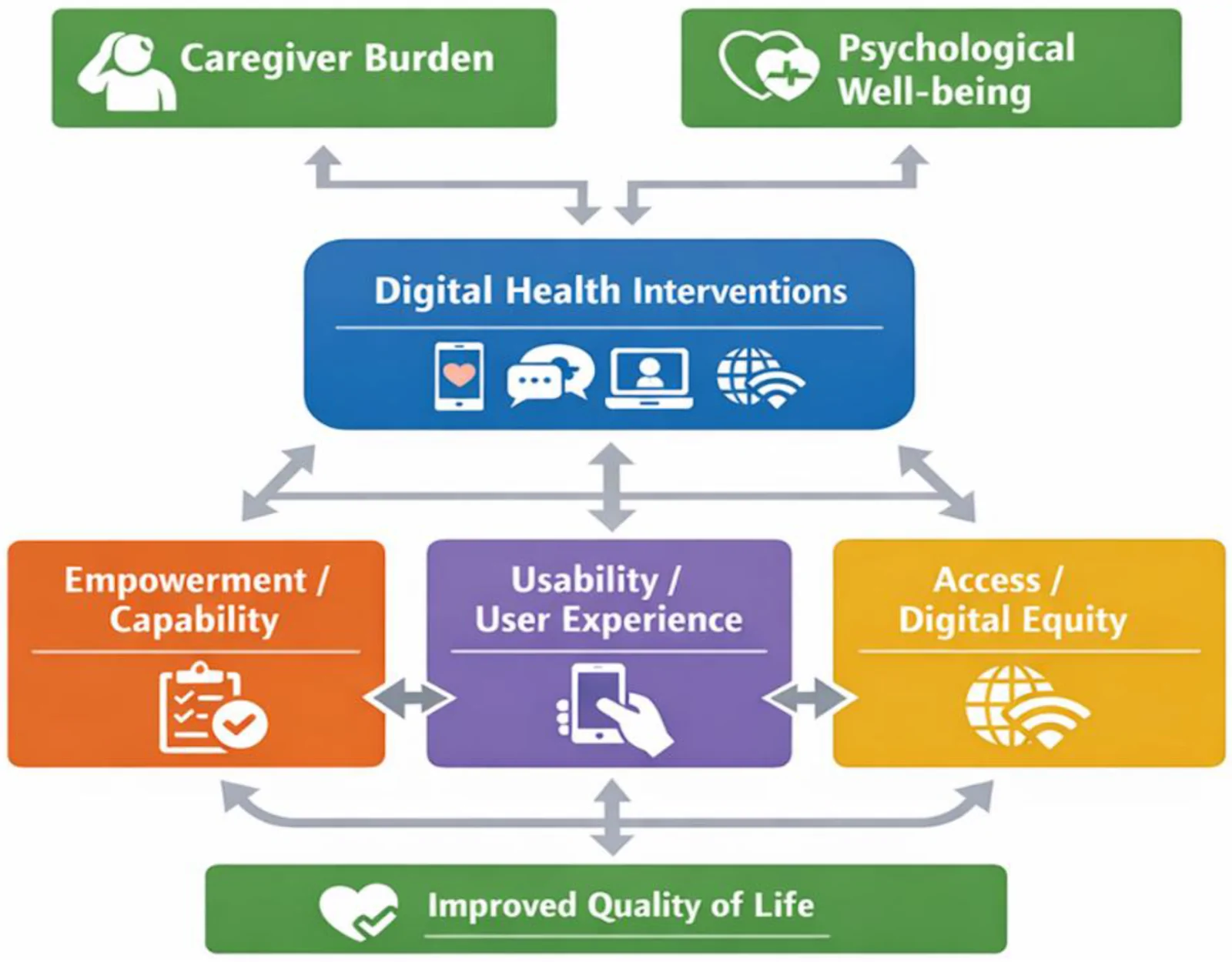
Conceptual model of how digital health interventions influence quality of life (QoL) among family caregivers of stroke survivors.

The potential impact of these interventions appears to depend on three interacting implementation mechanisms: caregiver capability, usability, and digital equity. Increased caregiver capability may improve confidence and preparedness; effective usability may enhance engagement; and equitable access may determine whether caregivers can benefit from digital resources.

However, because current evidence remains heterogeneous and frequently based on feasibility or preliminary studies, the conceptual model should be interpreted as a framework for understanding possible mechanisms rather than a confirmed causal pathway.

## 4 Discussion

This scoping review mapped the emerging evidence on digital health interventions designed to support family caregivers of stroke survivors and examined their relationship with caregiver-centered outcomes. Overall, available evidence suggests that digital interventions, particularly caregiver education, structured follow-up, and skills-building approaches, may support caregiver preparedness, perceived capability, and selected quality-of-life-related outcomes. However, the current evidence should be interpreted cautiously because confidence regarding intervention effectiveness remains limited due to substantial heterogeneity in intervention characteristics, outcome measures, follow-up duration, and methodological rigor among included studies.

A key contribution of this review is the distinction between direct caregiver-focused evidence and indirect caregiver-relevant evidence. Direct caregiver interventions, including caregiver education platforms, mobile applications, and online support programs, more consistently evaluated outcomes such as caregiver burden, psychological well-being, confidence, and caregiving capability. In contrast, patient-oriented or health-system-level digital interventions may generate potential caregiver benefits through improved continuity of care, reduced healthcare utilization, and enhanced home management; however, these benefits cannot be assumed unless caregiver outcomes are directly measured. This distinction is particularly important because many digital stroke interventions remain primarily patient-centered, while caregiver outcomes are frequently secondary or exploratory.

The findings indicate that intervention characteristics may influence potential caregiver benefits. Digital interventions combining structured educational content with professional support, such as nurse-guided messaging, telehealth follow-up, or interactive platforms, appeared more aligned with caregiver needs compared with passive information-only resources. Interactive interventions may enhance caregiver engagement by providing opportunities for feedback, clarification, and individualized support. Nevertheless, the available evidence is insufficient to determine whether specific delivery approaches, such as synchronous versus asynchronous communication or mobile applications versus web-based platforms, produce superior caregiver outcomes.

Several studies demonstrated promising findings regarding caregiver burden and capability. Educational interventions delivered through WhatsApp, mobile applications, and web-based platforms were associated with improvements in caregiver knowledge, confidence, and perceived ability to manage stroke-related care tasks (2, 5, 6). However, these improvements were more consistently observed in task-related caregiving outcomes than in broader quality-of-life domains. Differences in measurement instruments, intervention duration, and study design limit the ability to draw firm conclusions regarding sustained effects on caregiver quality of life.

Psychological outcomes represent another important but underdeveloped area. Some digital interventions demonstrated potential reductions in caregiver stress and improvements in emotional support; however, few studies specifically targeted the psychological needs of stroke caregivers. Many interventions were adapted from broader caregiver populations rather than developed specifically for stroke caregiving contexts. Future caregiver-centered digital interventions should incorporate psychological support components, particularly considering the prolonged emotional and social demands experienced by stroke caregivers.

The role of usability, digital literacy, and accessibility emerged as critical determinants of intervention engagement. Although digital health interventions provide opportunities to extend caregiver support beyond traditional healthcare settings, their benefits depend on whether caregivers can access, understand, and consistently use these technologies. Studies from Malaysia, China, Singapore, and Saudi Arabia highlighted challenges related to technology confidence, affordability, connectivity, privacy concerns, and the credibility of online health information (1, 4, 21). These findings emphasize that digital health interventions should be designed according to principles of health equity rather than assuming universal digital readiness.

At the health-system level, integrated mHealth models such as the SINEMA intervention demonstrated important clinical and economic benefits in stroke management, including improved patient outcomes and reduced recurrence-related outcomes (9, 10, 22). However, these findings primarily represent patient and health-system outcomes rather than direct evidence of improved caregiver quality of life. Therefore, while such models may indirectly support caregivers by improving care coordination and reducing caregiving complexity, future evaluations should include caregiver-specific outcomes to determine their true caregiver impact.

The findings also highlight the importance of participatory and adaptive design in digital caregiver interventions. Involving caregivers, stroke survivors, and healthcare professionals during development may improve usability, acceptability, and contextual relevance. Co-designed interventions should consider caregiver preferences, cultural factors, health literacy, privacy concerns, and resource limitations. Digital health solutions should therefore be viewed as complex healthcare interventions requiring alignment between technology, users, healthcare professionals, and existing care pathways.

### 4.1 Implications for clinical practice and digital health implementation

The findings suggest several implications for clinical practice, particularly for nurses and healthcare professionals involved in stroke rehabilitation and transitional care. Digital interventions should be considered as complementary tools that extend, rather than replace, human-centered caregiver support. Structured digital education, reminders, communication platforms, and remote follow-up may strengthen caregiver preparedness and confidence, particularly after hospital discharge when caregivers often experience uncertainty regarding stroke management.

Healthcare professionals should assess caregiver readiness before implementing digital interventions, including digital literacy, access to devices, internet availability, privacy concerns, and individual preferences. A blended approach combining digital tools with periodic professional interaction may be more appropriate than fully automated interventions, particularly among older caregivers or those with limited technological experience.

For digital health developers and policymakers, usability, accessibility, and equity should be considered fundamental components of intervention design rather than secondary considerations. Future digital caregiver interventions should prioritize:

1. simplified and user-centered interfaces;
2. culturally appropriate and understandable health information;
3. privacy and data security safeguards;
4. low-bandwidth functionality for resource-limited settings;
5. integration with existing stroke care pathways;
6. Training and technical support for caregivers.

Implementation strategies should also address disparities related to socioeconomic status, geography, age, and digital literacy. Without targeted support, digital health interventions may unintentionally widen existing inequalities by primarily benefiting caregivers who already have greater technological resources.

### 4.2 Implications for future research

Future research should move beyond feasibility evaluation and prioritize rigorous caregiver-centered trials assessing long-term outcomes. Studies should incorporate validated caregiver measures, including caregiver burden, psychological well-being, self-efficacy, preparedness, and quality-of-life-related outcomes.

Researchers should also provide more detailed reporting of intervention characteristics, including:

- target users (caregiver, patient-caregiver dyad, or healthcare system);
- intervention intensity;
- duration;
- degree of personalization;
- synchronous or asynchronous communication;
- healthcare professional involvement;
- usability testing;
- implementation outcomes.

Future studies should additionally evaluate cost-effectiveness, scalability, cultural adaptation, and strategies to improve accessibility among underserved caregiver populations.

The heterogeneity observed across included studies reflects the evolving nature of digital health interventions in stroke caregiving. Differences in intervention objectives, technological platforms, degree of healthcare professional involvement, caregiver characteristics, and outcome measurement approaches limit direct comparison between studies. Therefore, the findings should be interpreted as identifying promising intervention characteristics and implementation considerations rather than establishing comparative effectiveness between digital approaches.

## 5 Limitations of the evidence

This scoping review has several limitations. First, the restriction to English-language publications may have excluded relevant evidence from non-English-speaking settings, limiting global representation. Second, the inclusion of feasibility studies, protocols, and early-stage interventions was appropriate for mapping an emerging field but limits conclusions regarding intervention effectiveness and long-term caregiver outcomes.

Third, substantial heterogeneity was observed across intervention modalities, target users, outcome instruments, and follow-up periods, preventing quantitative comparison or meta-analysis. Fourth, included studies may have overrepresented caregivers with greater digital readiness and may underrepresent populations experiencing digital exclusion, such as older caregivers, rural communities, and individuals with limited financial or technological resources.

Finally, because this review did not perform formal risk-of-bias assessment, the findings should be interpreted as evidence mapping rather than confirmation of intervention effectiveness. Future systematic evaluations should incorporate methodological quality assessment and more standardized outcome reporting.

## 6 Conclusion

This scoping review indicates that digital health interventions may support family caregivers of stroke survivors by improving caregiver education, preparedness, access to support, and selected caregiver-related outcomes, including burden, psychological well-being, and caregiving capability. However, the current evidence remains heterogeneous, with substantial variation in intervention modalities, intensity, outcome measurements, follow-up periods, and study designs. Therefore, available evidence is insufficient to establish definitive effectiveness of digital health interventions in improving caregiver quality of life.

Digital interventions that incorporate structured education, professional support, user-centered design, and integration within existing stroke care pathways appear promising for enhancing caregiver support. Nevertheless, these findings should be interpreted as evidence of potential benefit rather than confirmation of causal effects. Future research should prioritize well-designed, multi-center randomized controlled trials with longer follow-up periods, standardized caregiver-centered outcome measures, and more inclusive recruitment strategies that address digital literacy, accessibility, and socioeconomic barriers.

## Data Availability

The original contributions presented in the study are included in the article/Supplementary Material, further inquiries can be directed to the corresponding author.
